# Development of a Submillimetric GNSS-Based Distance Meter for Length Metrology †

**DOI:** 10.3390/s21041145

**Published:** 2021-02-06

**Authors:** Luis García-Asenjo, Sergio Baselga, Chris Atkins, Pascual Garrigues

**Affiliations:** 1 Departamento de Ingeniería Cartográfica, Geodesia y Fotogrametría, Universitat Politècnica de València, Camino de Vera s/n, 46022 Valencia, Spain; lugarcia@cgf.upv.es (L.G.-A.); pasgarta@cgf.upv.es (P.G.); 2 Department of Civil, Environmental and Geomatic Engineering, University College London, Gower Street, London WC1E 6BT, UK; c.atkins@ucl.ac.uk

**Keywords:** Global Navigation Satellite Systems (GNSS), length, metrology, multipath

## Abstract

Absolute distance determination in the open air with an uncertainty of a few tenths of a millimetre is increasingly required in many applications that involve high precision geodetic metrology. No matter the technique used to measure, the resulting distances must be proven consistent with the unit of length (SI-metre) as realized in the outdoor facilities traditionally used in length metrology, which are also known as calibration baselines of reference. The current calibration baselines of reference have distances in the range of 10 to 1000 m, but at present there is no solution on the market to provide distances with submillimetric precision in that range. Consequently, new techniques such as multi-wave interferometry, two-wave laser telemeters or laser trackers are being developed. A possible alternative to those sophisticated and expensive techniques is the use of widely used Global Navigation Satellite Systems (GNSS) in order to provide a GNSS-Based Distance Meter (GBDM). The use of a GBDM as a potential technique for length metrology has been thoroughly analysed in several European research projects by using the state-of-the-art geodetic software, such as Bernese 5.2, but no definite conclusions have been drawn and some metrological questions are considered still open. In this paper, we describe a dedicated approach to build up a submillimetric GBDM able to be applied in the current calibration baselines of reference, as well as possible methods to cope with the multipath error of the GNSS signals which is the major limitation for the practical uptaking of the technique in metrology. The accuracy of the proposed approach has been tested following the length metrology standards in four experiments carried out in the Universitat Politècnica de València (UPV). The results demonstrate that the proposed GBDM can provide an accuracy of a few tenths of a millimetre in the current calibration baselines of reference.

## 1. Introduction

Absolute distance determination in the open air with an uncertainty of a few tenths of a millimetre is increasingly required in fields such as length metrology, high precision geodetic metrology, deformation monitoring at critical sites or local ties in Fundamental Geodetic Observatories (FGOs) [1,2,3].

In the present context, the term absolute refers to the fact that distances have to be consistent with the metre, the unit of length of the International System of Units (SI) which is currently defined as the length of the path travelled by light in vacuum during a time interval of 1/299792458 of a second [4]. This definition of SI-metre is realized by the National Metrology Institutes (NMIs) with a standard uncertainty $U_{\left(k=1\right)}=10^{-12}$ by using laser interferometers with stabilised frequencies, though such a high accuracy can only be obtained for short distances, i.e., several metres, under laboratory conditions. Following the core concept of metrological traceability, those references or primary standards are subsequently transferred or disseminated from laboratories to outdoor metrological facilities that are also known as calibrations baselines. These calibration baselines, whose inter-pillar lengths usually range from 10 m to 1000 m, are then used as secondary and working standards for practical purposes [5,6,7].

The only calibration baseline that has proved stable with a relative standard uncertainty near $u_{\left(k=1\right)}=10^{-8}$ for more than 70 years is the Nummela Standard Baseline (NSB) in Finland (864 m), and thus acknowledged as international standard in geodetic metrology. Such a high accuracy can only be obtained by using the Väisäla interferometer which has the additional metrologic advantage of being directly traced to SI-metre [8,9,10]. With this method being very demanding, the NSB inter-pillar distances are measured only every five years by the Finnish Geospatial Research Institute (FGI), although its accurate scale can be traceably transferred to other baselines of reference by using submillimetric Electronic Distance Meters (EDM) like the Kern Mekometer ME5000 (ME5000) which was manufactured in the late 1980s [11,12].

Nevertheless, the use of the out-of-production ME5000 entails two problems. Firstly, only a few units of this type of EDM are still in use, and secondly, having only one carrier phase (0.628 nm), the air refractivity has to be determined with an accuracy better than 0.1 ppm by using meteorological sensors, which is rather difficult for distances longer than 1 km [12]. Consequently, new techniques such as multi-wave interferometry, two-wave laser telemeters or laser trackers are currently required to go beyond the ME5000 [13,14,15]. Yet the research effort is considerable, and it demands joint projects like the current 18SIB01 GeoMetre—large-scale dimensional measurements for geodesy’ which has received funding from the EMPIR programme co-financed by the participating states and from the European Union’s Horizon 2020 research and innovation programme [16].

A possible alternative to those expensive techniques is the use of Global Navigation Satellite Systems (GNSS) to develop a GNSS-Based Distance Meter (GBDM). Some experiments that compare GNSS distances with EDM distances can be found in the literature, but being most of them based in the use of commercial GNSS software and EDM instruments, the differences between these two techniques can easily reach around 3 mm [17,18]. The GBDM as a potential technique for length metrology was firstly analysed in the EMRP-JRP 3.1-TP 3 ‘Length’ [19], and subsequently in the EMRP-JRP SIB60 ‘Surveying’ [20]. Most of the studies were performed by using state-of-the-art geodetic software, i.e., the Bernese 5.2 [21], along with the most advanced geodetic products and models. The reporting document for the GBDM technique recommends the use of carrier-phase double differences (DD), and points out that GNSS signal multipath is the major limiting factor to get submillimetric accuracy, but no recommendation is given concerning a possible standard strategy for optimal GNSS processing and some metrological questions such as uncertainty determination or traceability are considered still open [22]. Consequently, some criticism still remains for the GNSS to be used as a submillimetric technique for length metrology.

However, previous research conducted at the Department of Cartographic Engineering, Geodesy and Photogrammetry of the *Universitat Politècnica de València* (DICGF-UPV) showed the potential of GNSS techniques in terms of reproducibility for measuring distances over several hundred metres with uncertainties below one millimetre by using a functional model different from the traditional geodetic approach along with robust estimation [1,23,24]. The initial assumptions, the functional models and the intended computing approach are described in Section 2.

As previously mentioned, the GNSS signal multipath error is considered the major limitation to use a GBDM in current metrologic facilities. The traditional geodetic approach to cope with this problem is to carefully select the GNSS sites and average out the multipath error by using long observation span times. However, current calibration baselines of reference [10,25] were mostly set up decades ago, and they do not always have favourable conditions for GNSS measurements since they are normally surrounded by trees or buildings. As a result, L1/E1 DD residuals can easily reach up to several centimetres with no Gaussian distribution and consequently the obtained distance will be biased, thus impeding the desired submillimetric accuracy. Therefore, a consistent GBDM should include some processing strategy for multipath mitigation. Therefore, the last field experiment was specially designed to investigate and develop possible methods for multipath mitigation as applied to length metrology. This part of the investigation was conducted at the Space Geodesy and Navigation Laboratory of the University College London (SGNL-UCL), and their results are shown in Section 3.

Nonetheless, not only does the GBDM technique have to be proven to be precise but it must also be accurate according to length metrology standards. Thus, the proposed GBDM technique was tested in four field experiments conducted at the UPV submillimetric calibration baseline from the years 2013 to 2016. Each experiment was a four-day campaign where both the GNSS and EDM techniques were applied. During the daylight hours, the inter-pillar distances of the calibration baseline were measured using a ME5000 in accordance to ISO17123-4 [5], and subsequently, GNSS antennas were set up to collect twelve-hour sessions overnight. The same operational scheme was repeated for four consecutive days. The experiment was designed in such a way that the obtained distances can be traced to the SI-metre.

This paper is organised as follows. In Section 2, the general approach for GNSS data processing is described. Section 3 is devoted to the methods for multipath detection and mitigation that were developed and tested. The assessment of the resulting accuracy by comparison with distances derived from EDM traced to the SI-metre definition in a calibration baseline is explained in Section 4. Finally, some conclusions and future work are summarised in Section 5.

## 2. GNSS Data Processing Approach

### 2.1. Basic Carrier-Phase Equation

Unlike the traditional EDM distance determination, where electromagnetic signal path travels several hundred metres between both ends of the measured baseline, the GNSS-based distance has to be indirectly obtained from electronic signals that travel from satellites to the antennas which involves distances around 22,000 km. The general equation for a carrier phase observable can be written as follows

(1) $$\begin{array}{c} l\Phi_{i}^{k}\left(t\right)=\rho_{i}^{k}\left(t_{i}\right)+\left[c-{\dot{\rho }}_{i}^{k}\left(t_{i}\right)\right]dt_{i}-cdt^{k}-I_{i,\Phi }^{k}\left(t\right)+T_{i}^{k}\left(t\right)+\lambda N_{i}^{k}\\ +\left[\Phi_{i}\left(t_{0}\right)-\Phi^{k}\left(t_{0}\right)\right]+\delta_{i}+\delta^{k}+\delta m_{i,\Phi }^{k}\left(t\right)+\lambda \epsilon_{i}^{k}\left(t\right) \end{array}$$

where:
$\Phi_{i}^{k}\left(t\right)$carrier phase measurement collected at receiver time $t_{i}$, in m$\rho_{i}^{k}\left(t\right)$geometric distance between the receiver *i* and the satellite *k*, in m*c*speed of light in vacuum, in m/s${\dot{\rho }}_{i}^{k}\left(t\right)$topocentric range rate of the satellite *k* with regard to the receiver *i*, in m/s$dt_{i}$receiver clock offset, in s$dt^{k}$satellite clock offset, in s$I_{i,\Phi }^{k}\left(t\right)$ionospheric delay evaluated at epoch $t_{i}$, in m$T_{i}^{k}\left(t\right)$tropospheric delay evaluated at epoch $t_{i}$, in mλcarrier phase wavelength, in m$N_{i}^{k}$integer ambiguity for the carrier phase observable, in cycles$\Phi_{i}\left(t_{0}\right)$initial carrier phase offset for the receiver *i*, in m$\Phi^{k}\left(t_{0}\right)$initial carrier phase offset for the satellite *k*, in m$\delta_{i}$receiver hardware delay, in m$\delta^{k}$satellite hardware delay, in m$\delta m_{i}^{k}\left(t\right)$carrier phase multipath error, in m$\epsilon_{i}^{k}\left(t\right)$random noise, in cycles

For the sake of simplicity, the notation assumes the use of only one constellation (e.g., GPS).

### 2.2. Initial Assumptions

Our approach to investigate the capabilities of GNSS for length determination with submillimetric accuracy relies on four initial assumptions: First, modern receivers provide carrier phase observations with submillimetre level noise. Secondly, distances determined by GNSS are highly stable at a global scale. Thirdly, the modulus of a vector between two GNSS receiver antennas can be more precisely determined than its corresponding components. Finally, the increasing number of operational GNSS satellites allows one to optimise the selection of satellites to improve length determination. These four assumptions are discussed below.

**Figure 1:**
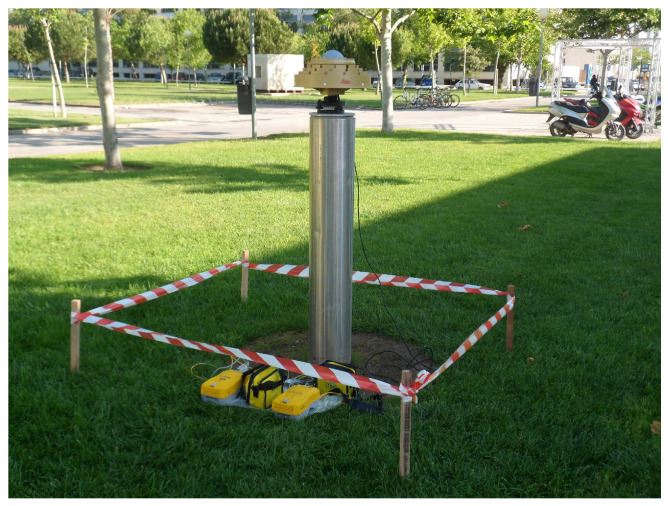
Global Navigation Satellite System (GNSS) measurement at the Universitat Politècnica de València (UPV) calibration baseline using a 3D choke-ring antenna equipped with a signal splitter and two receivers collecting the same signals.

Regarding the noise of the GNSS carrier phase signal, it used to be assumed as a random error of 1% of the carrier wavelength, but recent literature shows that modern GNSS receivers can provide enhanced performance [26,27]. The random noise of a GNSS signal depends on the electronics of the receivers, but also on the quality of the signal. Therefore, a different noise should be expected for each constellation and even for each individual satellite. A standard method to assess the overall random noise of a GNSS receiver is to perform a zero baseline test, which consists of processing the double differences of phase observations of two receivers that are connected to the same antenna by means of a signal splitter (see Figure 1).

**Figure 2:**
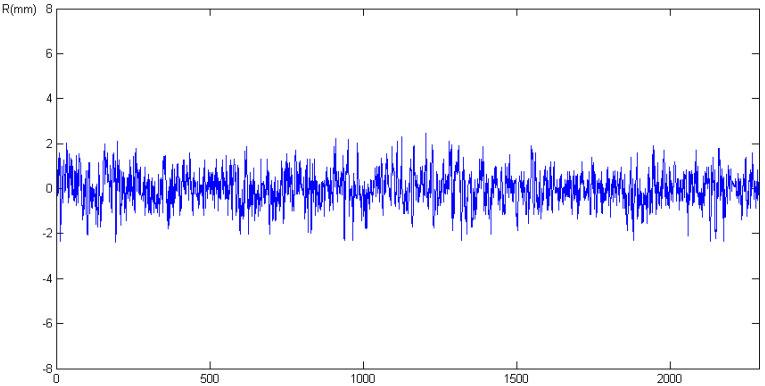
Zero-baseline double difference residuals for a pair of multi-constellation Leica GS10 in July 2015.

Figure 2 shows the double difference residuals obtained using two multi-constellation Leica GS10 receivers. The overall root mean square (RMS) value obtained for L1 double difference residuals was 0.7 mm, thus confirming our first initial assumption that they are currently below 1 mm × 2, that is, as we assumed, less than 1 mm of standard error for a carrier phase observation amplified by a factor of 2 due to the double difference combination (see e.g., expression (20.67) in Reference [28]).

Concerning the GNSS scale stability, it is maintained worldwide at the level of 1 ppb $=10^{-9}$, which is to say 1 mm over 1000 km. Nonetheless, some open questions still remain as to whether this high absolute scale accuracy is also applicable to short distance determination in the existing metrological infrastructures. Moreover, the GNSS scale stability relies on the accuracy of the precise ephemeris and ultimately on the International Terrestrial Reference Frame (ITRF) which is used for their determination. Since the ITRF scale is determined by a combination of Satellite Laser Ranging (SLR) and Very Long Baseline Interferometry (VLBI) observations whose scale differs by about $10^{-9}$ [29], the linkage between the centre of the instruments used by SLR, VLBI and GNSS at Fundamental Geodetic Observatories (FGO), also known as local ties, should be performed with submillimetric accuracy [2]. Thus, an additional benefit of submillimetric length determination with an appropriate metrological uncertainty evaluation based on traceable instruments and methods would be to improve the ITRF scale and consequently the accuracy of the precise ephemeris of GNSS satellites.

The third assumption is that GNSS distance may be better determined than the individual components of the corresponding vector. Double difference processing usually yields precise vectors whose components ΔX,ΔY,ΔZ are subsequently used to compute the distance

(2) $$D=\sqrt{\Delta X^{2}+\Delta Y^{2}+\Delta Z^{2}}$$

Fortunately, correlation usually leads to $\sigma_{D}<\sqrt{\sigma_{\Delta X}^{2}+\sigma_{\Delta Y}^{2}+\sigma_{\Delta Z}^{2}}$ (and even $\sigma_{D}<\sigma_{\Delta X}$ and $\sigma_{D}<\sigma_{\Delta Y},\sigma_{D}<\sigma_{\Delta Z}$) because in the expression

(3) $$\begin{array}{ccc} \sigma_{D}^{2} & = & {\left(\frac{\Delta X}{D}\right)}^{2}\sigma_{\Delta X}^{2}+{\left(\frac{\Delta Y}{D}\right)}^{2}\sigma_{\Delta Y}^{2}+{\left(\frac{\Delta Z}{D}\right)}^{2}\sigma_{\Delta Z}^{2}\\ & + & 2\frac{\Delta X}{D}\frac{\Delta Y}{D}\sigma_{\Delta X\Delta Y}+2\frac{\Delta X}{D}\frac{\Delta Z}{D}\sigma_{\Delta X\Delta Z}\\ & + & 2\frac{\Delta Y}{D}\frac{\Delta Z}{D}\sigma_{\Delta Y\Delta Z} \end{array}$$

the covariances may be positive or negative depending on the relative geometry between the baseline and the observed satellites. Thus, a proper selection of observables may lead to a reduction in the standard error of the obtained GNSS distance.

Finally, a multi-constellation approach has clear advantages for length determination if compared to a single-constellation one, i.e., only GPS. A large number of satellites in view allows the inclusion in the system of normal equations of only those double differences whose impact on the baseline is lower than an assumed threshold as well as to eliminate those satellites whose phase observables contain a strong multipath error. As an illustration, in the last GNSS campaign that was carried out at the UPV calibration baseline in July 2015, the number of satellites registered in a time span of eight hours was the following: 21 GPS, 19 GLONASS and 4 Galileo. Thus, the inclusion of the new and emerging constellations gives rise to new possibilities for precise GNSS length determination.

### 2.3. Functional Model for the Initial Coordinates

The GBDM technique starts with the determination of the absolute coordinates of the ends of the measured distance. This initial solution has to provide three-dimensional coordinates with an accuracy of a few centimetres in the same coordinate reference frame as the precise ephemerides used in the second step. This requirement is not very difficult to fulfil, and can be achieved by using different strategies, but we recommend the use of a common Precise Point Position (PPP) solution. This approach, which is based on an epoch-wise processing strategy that uses an ionosphere-free combination with floating ambiguities, is relatively easy to implement or can be alternatively obtained from online services like CSRS-PPP [30].

Some advantages of this straightforward approach are that the bulk of the ionosphere error is removed, slow changes of the tropospheric delays can be easily absorbed by parametrization, the receiver clock offsets are well determined and the obtained ionosphere-free residuals are expected to be mostly multipath error and possible antenna calibration mismodelling.

Concerning the functional model, a widely used equation for receiver *i* and satellite *k* is (see, e.g., [31])

(4) $$\begin{array}{cc} h_{\Phi_{IF}}= & \rho_{i}^{k}+c\delta_{i}+A_{i}^{k}+m_{G}\left(\theta_{i}^{k}\right)T_{zwd}\\ \; & +m_{G}\left(\theta_{i}^{k}\right)\cot\left(\theta_{i}^{k}\right)\left[G_{N}\cos\psi_{i}^{k}+G_{E}\sin\psi_{i}^{k}\right] \end{array}$$

where:
$h_{\Phi_{IF}}$ionosphere-free combination (m)$\rho_{i}^{k}$modelled geometric range (m)*c*speed of light in vacuum (m/s)$\delta_{i}$receiver clock offset (s)$A_{i}^{k}$non-integer ambiguity term (m)$\theta_{i}^{k},\psi_{i}^{k}$elevation and azimuth angles (rad)$T_{zwd}$zenith wet troposphere delay (m)$m_{G}\left(\right)$Global Mapping Function (GMF)$G_{N},G_{E}$north and east troposphere gradients

The non-integer ambiguity term $A_{i}^{k}$ comprises the integer ambiguity, the initial phase offsets for both frequencies, and the hardware delays. The vector of Kalman filter states that are estimated is

(5) $$\vec{x}={\left(X_{i}\;Y_{i}\;Z_{i}\;c\delta_{i}\;T_{zwd}\;G_{N}\;G_{E}\;A_{i}^{1}\cdots A_{i}^{m}\right)}^{T}$$

Taking into account the implemented PPP approach, the obtained ionosphere-free residuals are expected to be mostly multipath error and possible antenna calibration mismodelling [32].

### 2.4. Functional Model for the Double Differences

The functional model used in our approach is specifically tailored to length determination and differs from the corresponding geodetic approach in four ways: only L1/E1 double differences are processed, ambiguity determination is not required, and both ionospheric and tropospheric corrections are not included.

Since L1/E1 carrier phase observables are less noisy than L2, L5, E5a or E5b observables, or any combination of them [33], double differences of L1/E1 carrier phase are the preferred equations to obtain the final solution.

A free ambiguity approach reduces the number of unknowns to determine thus strengthening the model [34]. The only requirement for using the free-ambiguity method is a prior determination of the absolute coordinates of both ends with an accuracy of a few centimetres, which can be achieved by means of a PPP static processing, thus obtaining $\left(X_{i}^{\left(0\right)},Y_{i}^{\left(0\right)},Z_{i}^{\left(0\right)}\right)$ and $\left(X_{j}^{\left(0\right)},Y_{j}^{\left(0\right)},Z_{j}^{\left(0\right)}\right)$

Given the double difference equation

(6) $$\lambda \varphi_{ij}^{kl}=\rho_{ij}^{kl}+\lambda N_{ij}^{kl}+\epsilon_{ij}^{kl}$$

for substraction with the order

(7) $$\varphi_{ij}^{kl}=\left(\varphi_{j}^{l}-\varphi_{i}^{l}\right)-\left(\varphi_{j}^{k}-\varphi_{i}^{k}\right)=\varphi_{j}^{l}-\varphi_{i}^{l}-\varphi_{j}^{k}+\varphi_{i}^{k}$$

and expanding around $\left(X_{j}^{\left(0\right)},Y_{j}^{\left(0\right)},Z_{j}^{\left(0\right)}\right)$ with $\left(X_{i}^{\left(0\right)},Y_{i}^{\left(0\right)},Z_{i}^{\left(0\right)}\right)$ held fixed, Equation (6) can be rewritten as

(8) $$\begin{array}{ccc} \frac{\lambda \varphi_{ij}^{kl}-\rho_{ij}^{kl}}{\lambda } & = & \frac{1}{\lambda }\frac{\partial \rho_{ij}^{kl}}{\partial X_{j}}dX_{j}+\frac{1}{\lambda }\frac{\partial \rho_{ij}^{kl}}{\partial Y_{j}}dY_{j}\\ & + & \frac{1}{\lambda }\frac{\partial \rho_{ij}^{kl}}{\partial Z_{j}}dZ_{j}+N_{ij}^{kl}+\frac{1}{\lambda }\epsilon_{ij}^{kl} \end{array}$$

which can be subsequently arranged as

(9) $$\begin{array}{ccc} \frac{\lambda \varphi_{ij}^{kl}-\rho_{ij}^{kl}}{\lambda }-int\left(\frac{\lambda \varphi_{ij}^{kl}-\rho_{ij}^{kl}}{\lambda }\right) & = & \frac{1}{\lambda }\frac{\partial \rho_{ij}^{kl}}{\partial X_{j}}dX_{j}+\\ +\frac{1}{\lambda }\frac{\partial \rho_{ij}^{kl}}{\partial Y_{j}}dY_{j}+\frac{1}{\lambda }\frac{\partial \rho_{ij}^{kl}}{\partial Z_{j}}dZ_{j} & + & \frac{1}{\lambda }\epsilon_{ij}^{kl} \end{array}$$

where the only unknowns to be solved are $dX_{j},dY_{j},dZ_{j}$.

Regarding tropospheric delays, our current model assumes that they are negligible for distances below 1 km. Note that existing metrological facilities are small and points of interest are usually at about the same altitude. Consequently, the path taken by a GNSS signal through the atmosphere to each end of a baseline is almost the same, especially for those satellites with higher elevation. In addition, we collected GNSS data in the nighttime. Thus, only residual atmospheric delay errors with minor influence on the distance are expected.

For longer distances, ionospheric delay can be determined using a combination of carrier waves [28] and subsequently use them to correct L1/E1 carrier phase observables. On the contrary, the influence of residual tropospheric delays should be taken into account when both ends of the measured baseline have a substantial height difference which can happen in some local ties or geodetic control networks. Since the tropospheric errors expected in the double difference equations are very small and also dependant on local meteorological parameters, global models may not be accurate enough for submillimetric length determination.

## 3. GNSS Multipath Mitigation

The GNSS multipath error arises from the fact that the signal from a satellite arrives at the antenna via multiple paths due to reflection and diffraction. These indirect-path signals can distort the received signal by up to one quarter-cycle and this error does not cancel or mitigate when forming the double difference combination. Therefore, GNSS multipath becomes a critical source of error in high precision applications [28,35], and even more for length metrology because most of the current calibration baselines of reference, which were set up decades ago before the advent of the space geodesy era, are surrounded by objects such as trees, buildings or other facilities.

Multipath interference can be separated into near-field and far-field effects, which do have different properties. Near-field multipath results from the closest vicinity of the antenna, i.e., the first 50 cm around the antenna, and mainly leads to a systematic bias that can be prevented by creating identical near-field situation in all stations which involves the same type of pillars, GNSS antenna model, antenna mounting, and routing of the antenna cable. However, the near-field can also change the electronic properties of the antenna and thus, individual antenna calibrations can be considered accurate as long as the actual near-field situation was reproduced during the calibration procedure. Nonetheless, this refinement is not usually included in the calibration process due to size and weight limitations and it would only make sense if each calibrated antenna was always setup in the same pillar [22,35,36,37].

On the other hand, far-field effects arise from reflecting surfaces in the environment of the antenna such as trees or buildings, and for static surveys, which is the case at hand, lead to short periodic errors. Following the traditional geodetic approach, the far-field multipath is generally assumed to average out by sufficiently long observation times [35,38,39]. In consequence, this approach is particularly valid for permanent GNSS stations which have a favourable observational environment and 24-h sessions but cannot always be applied to calibration baselines and short time spans. Our initial approach to cope with multipath was based on the use of the signal-to-noise ratio flag and global robust estimation [40].

**Figure 3:**
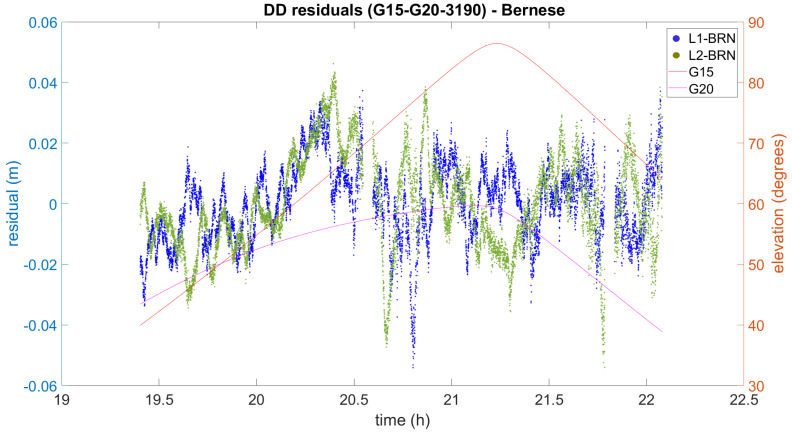
Example of double difference (DD) residuals that were obtained using Bernese 5.2.

Interestingly for our study, the trees surrounding the UPV calibration baseline, which were young in 2012, are increasing in size as the years go by. As a consequence, our former approach to cope with multipath, based on the use of the signal-to-noise ratio flag and global robust estimation [40], cannot be successfully applied under these new conditions. To give a picture of the problem, some DD residuals which were obtained in our experiment are plotted in Figure 3. It can be clearly seen that the L1 DD residuals range from 0.04 m to −0.05 m and they cannot be considered Gaussian. Therefore, the obtained distance will be biased. In addition, the L2 DD residuals are bigger, as expected, and follow a different pattern than the L1 DD residuals. Similar residuals were found in other DD combinations.

Over the last 10 years, the Space Geodesy and Navigation Laboratory of University College London (UCL) have developed and successfully applied techniques for GNSS multipath detection and mitigation, some of them suitable for GNSS processing for distance determination [31,32,41,42]. Specifically, we have now implemented the observation-domain sidereal filtering technique and the signal-to-noise ratio (SNR) estimator.

The sidereal filtering technique [31,32] takes advantage of the consecutive passes of each satellite to predict the multipath error. As long as both azimuth and elevation repeat for the same satellite, the multipath effect can be considered similar, and subsequently corrected. For GPS satellites, the repeating time is one sidereal day, which makes the technique attractive to use if data from consecutive days are available, whereas for Galileo satellites the repeating time increases to 10 sidereal days and the use of the technique would require GNSS observation sessions 10 days apart, which is impractical. In what follows, we will restrict the application of the sidereal filtering technique to GPS observations only.

In contrast, the SNR estimator does not provide a value for the multipath error [41]. By differencing SNR measurements on different frequencies and comparing the result with that obtained in a low-multipath environment, multipath can be detected. The result is a number that can be used, in theory, to identify the presence of multipath.

**Figure 4:**
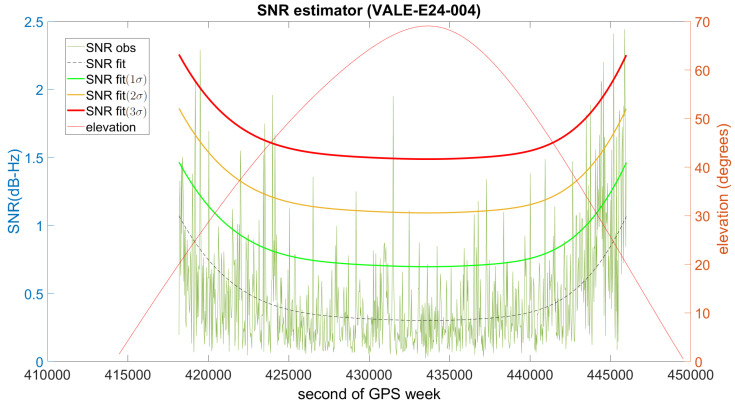
Low-multipath function used as reference to compute the SNR estimator for Galileo satellites.

To characterize a low-multipath environment, we opted for the VALE permanent station. It is only 100 m away from the UPV calibration baseline and its equipment (Leica GR10 receiver and Leica AR25.R3 antenna) is very similar to the equipment used in our experiment described in the next section. Figure 4 shows the SNR estimator for the Galileo satellite that was selected as a low-multipath reference. The line in black is the expected value, and in green, orange and red are respectively the 1σ, 2σ and 3σ thresholds. If the estimator is clearly above the red line, multipath is considered to be present.

Both techniques, sidereal filtering and SNR estimator, may be complementary. This is important since each technique may on certain occasions yield null values even when multipath is present. Conversely, depending on some aspects of the PPP implementation, e.g., cycle-slip detection and correction strategies, the residuals obtained could be abnormally high with no relation to multipath. However, matching residuals by using azimuth and elevation has clear advantages over time. First, the technique is independent of the value of multipath and does not need to rely on the identification of the correct satellite repeat period (a significant problem so far, as pointed out in Reference [43]). Second, the multipath model could be eventually parameterised in terms of azimuth and elevation even for the satellites with low multipath.

In summary, the whole process to mitigate multipath in the context of GBDM consists of the following four steps

1. To obtain a PPP solution for each site and for every day using an ionosphere-free combination.
2. To match the ionosphere-free observation residuals on successive days using azimuth and elevation in order to obtain a site-specific and time-specific multipath model for an ionosphere-free combination.
3. To form ionosphere-free DDs using the obtained multipath models with the same DD scheme that will be eventually used in the final L1/E1 solution.
4. To determine the L1/E1 DD multipath corrections from the previous values by using DD residuals from an independent DD solution and the same ionosphere-free combination that was used to obtain the PPP solution.

In the first step, the PPP solution is obtained with an epoch-wise processing strategy that uses an ionosphere-free combination with floating ambiguities. Some advantages of this straightforward approach are that the bulk of the ionosphere error is removed, the multipath error is amplified with respect to that of the original observed frequencies, and it can be implemented in real-time if desired.

Nevertheless, part of the multipath error could slip into other parameters and errors caused by the imperfect modelling of other parameters (receiver clock, troposphere delay, phase ambiguities) could be wrongly considered as multipath. For that reason, it is relevant to look for a repeating pattern in the second step. Only the residuals that follow a sidereal pattern can be considered as multipath error. Once the consecutive days are matched using azimuth and elevation, the median of the residuals is retained as multipath error. The result is a site-specific and time-specific multipath model for the combination used in the PPP solution. Interestingly, the model formed may contain some possible antenna calibration mismodelling since this error also repeats with azimuth and elevation.

In the third step, the site-specific and time-specific models that were built up for each satellite are combined to form the required ionosphere-free DD residuals. This combination largely cancels those components of the residuals which originate in the satellites, such as orbits and clocks, those related to the receiver clock offsets and possible residual atmospheric errors.

Finally, the L1/E1 DD multipath corrections are obtained for each day by using the ionosphere-free DD corrections and the corresponding DD residuals from an independent DD auxiliary solution. For that independent DD solution several frequencies such as L2, L5, E5a or E5b can be used as long as they were employed in the PPP solution. Obviously, the noise of the new multipath-corrected DD equation is higher then the original one, but that is not a problem for the case at hand as long as the multipath error is mitigated in such a way that the resulting distance is not biased.

The observation-domain sidereal filtering for GPS Precise Point Positioning (PPP) developed at the SGNL-UCL [31] uses the ionosphere-free linear combination $L_{3}$ with the following combination factors

$$\begin{array}{ccc} lll\alpha =\frac{77\lambda_{3}}{\lambda_{1}} & =\frac{f_{1}^{2}}{f_{1}^{2}-f_{2}^{2}} & =2.5457277801631\\ \beta =-\frac{60\lambda_{3}}{\lambda_{2}} & =-\frac{f_{2}^{2}}{f_{1}^{2}-f_{2}^{2}} & =-1.5457277801631 \end{array}$$

Please note we have chosen the notation $L_{3}$, which is used in the Bernese software, to be consistent with $L_{1}$ and $L_{2}$.

The formed $L_{3}$ combination has a wavelength

$$\lambda_{3}=\frac{\lambda_{1}\lambda_{2}}{77\lambda_{2}-60\lambda_{1}}≃0.006\;m$$

and with standard noise values $\sigma_{L_{1}}=\sigma_{L_{2}}=2$ mm, the resulting $L_{3}$ noise is

$$\sigma_{L3}=\sqrt{\alpha^{2}\sigma_{L_{1}}^{2}+\beta^{2}\sigma_{L_{2}}^{2}}=0.006\;m$$

Nonetheless, modern receivers can measure the carrier phase with better precision and from our experience the values $\sigma_{L_{1}}=\sigma_{L_{2}}=1$ mm are more realistic so that the resulting noise for the $L_{3}$ combination can be considered around 3 mm.

Since the noise of the ionosphere-free combination is $\sigma_{L3}\approx 3$ mm, the obtained DD residuals are expected to have a theoretical noise $\sigma_{DD_{L3}}\approx 6$ mm that could hamper the determination of a $L_{1}$ site-specific multipath model by using a PPP approach. Surprisingly, if $DD_{L1}$ residuals are obtained straight from the $DD_{L3}$ residuals by substracting the corresponding $DD_{L2}$ residuals, the resulting residuals have a noise $\sigma_{DD_{L1}}\approx 2$ mm. The reason is that the coefficients are smaller than one.

(10) $$L_{1}=\frac{1}{\alpha }L_{3}-\frac{\beta }{\alpha }L_{2}$$

and therefore,

(11) $$\sigma_{L_{1}}^{2}={\left(\frac{1}{\alpha }\right)}^{2}\sigma_{L_{3}}^{2}+{\left(\frac{\beta }{\alpha }\right)}^{2}\sigma_{L_{2}}^{2}\approx 2\;mm$$

**Figure 5:**
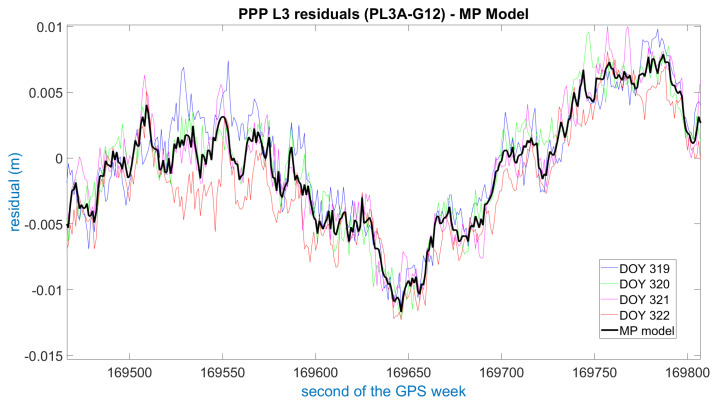
Example of Precise Point Positioning (PPP) residuals from four consecutive days after matching using azimuth and elevation. The GPS time is referred to the first day (2016 DOY 319). The obtained multipath model is shown in black.

Figure 5 illustrates the PPP ionosphere-free phase residuals for pillar 3 (PL3A) (see Figure 1) and satellite G12 after the matching process. As can be seen, the residuals from the four consecutive days of the experiment are consistent and show a clear sidereal pattern. Therefore, the initial assumption for the PPP residuals to be mostly multipath error is confirmed. When the residuals from the consecutive days are compared with each other, the differences are normally below 5 mm, thus confirming the correctness of the functional model used in the PPP implementation and its suitability for this application. Nevertheless, in some cases the PPP residuals differ by up to 1 cm, possibly due to a slightly different satellite trajectory, different humidity of the ground or technicalities of the particular PPP implementation. In those cases, there is still some consistency between days, and normally only one day at a maximum is different from the rest, so that retaining the median as the multipath value for the model usually solves the problem.

**Figure 6:**
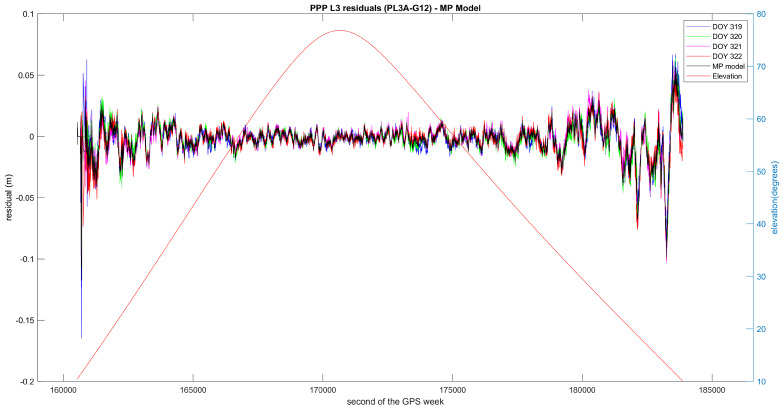
PPP residuals from four consecutive days after matching using azimuth and elevation for the entire observation session. The GPS time is referred to the first day (2016 DOY 319). The obtained multipath model is shown in black.

The figure above is a zoomed-in excerpt from Figure 6, which shows the residuals and the multipath model for the entire observation time for this particular satellite, that is, the approximately 6.5 h the satellite has been with an elevation above 10°. Evidently, the observations of low elevation are strongly affected by multipath and should not be used for precise positioning.

**Figure 7:**
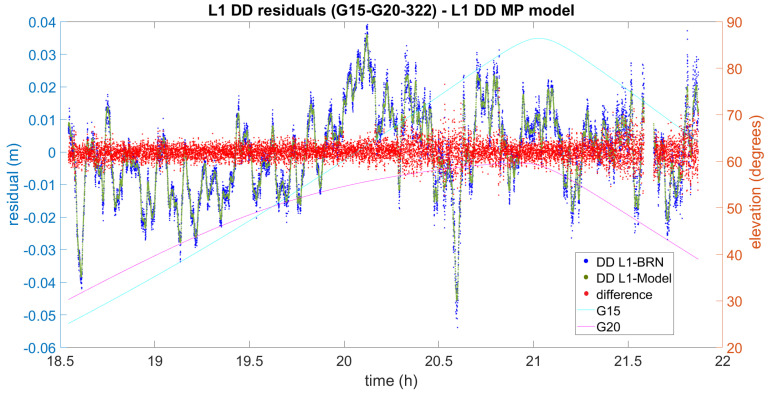
The result of L1 DD multipath mitigation for satellites G15 and G20 (2016 DOY322). DD L1-BRN and DD L1-Model denote the DD L1 residuals obtained by means of Bernese and the use of the Model, respectively.

**Figure 8:**
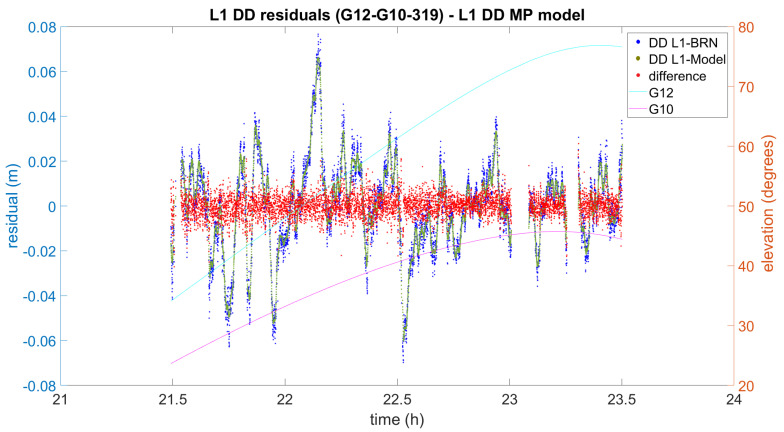
The result of L1 DD multipath mitigation for satellites G12 and G10 (2016 DOY319). DD L1-BRN and DD L1-Model denote the DD L1 residuals obtained by means of Bernese and the use of the Model, respectively.

**Figure 9:**
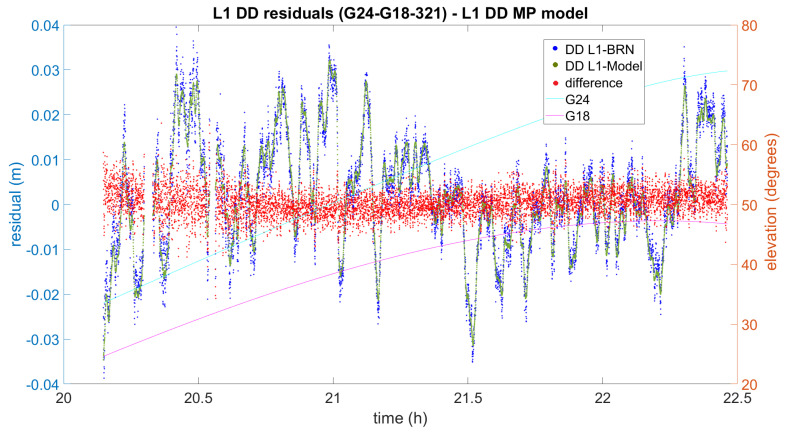
The result of L1 DD multipath mitigation for satellites G24 and G18 (2016 DOY321). DD L1-BRN and DD L1-Model denote the DD L1 residuals obtained by means of Bernese and the use of the Model, respectively.

Now let us focus on the impact on DD processing. In Figure 7, Figure 8 and Figure 9 the difference between the initial L1 DD residuals obtained from the Bernese solution (blue) and the L1 DD multipath correction given by the proposed multipath mitigation method (green) are plotted (red) for three different DD combinations and days. In the three examples, the L1 DD multipath error is mitigated and the new observed minus computed value is lower than one centimetre on average. The three examples also show that the proposed method seems to work independently of the elevation of the satellites, and even in the presence of data gaps.

As it can be seen Figure 7, the range of the obtained differences is 5 mm on average. However, some values at around 20.6 h have an absolute value of about 2 cm with residuals peaking −5 cm. Even in that case, the influence of the multipath error is largely diminished in the new observed-minus-computed-term, and the overall impact on the distance obtained can still be considered unbiased.

The example in Figure 8 shows higher residuals that reach up to 8 cm. In this case, the bandwidth of the differences is over 1 cm, and slightly higher if compared to the previous example. Again, the differences seem to be randomly distributed around zero and no impact is expected in the subsequent obtained distance.

Finally, the example given in Figure 9 has been chosen because the differences between the initial L1 DD residuals and the multipath corrections seem to be not so well centred as other DD combinations. In this case, although the overall range of the differences is over 8 mm, it can be seen that it is wider and slightly biased when both satellites are at a low elevation.

## 4. Assessment of the GNSS-Based Distances Accuracy

### 4.1. The UPV Calibration Baseline of Reference

To validate the accuracy of the GNSS-based distances they have to be compared with distances accurately traced to SI-metre in an outdoor metrological infrastructure. For practical reasons, we opted for the UPV calibration baseline. This baseline, which was set up in November 2007 by the Department of Cartographic Engineering, Geodesy and Photogrammetry (DICGF), was originally planned to evaluate the uncertainty of geodetic instruments according to ISO 17123 series [44]. In 2012, absolute scale was transferred from de Nummela Standard Baseline (NSB) by the Finnish Geospatial Research Institute (FGI) so as to carry out the experiment to validate the accuracy of the GNSS-based distances.

**Figure 10:**
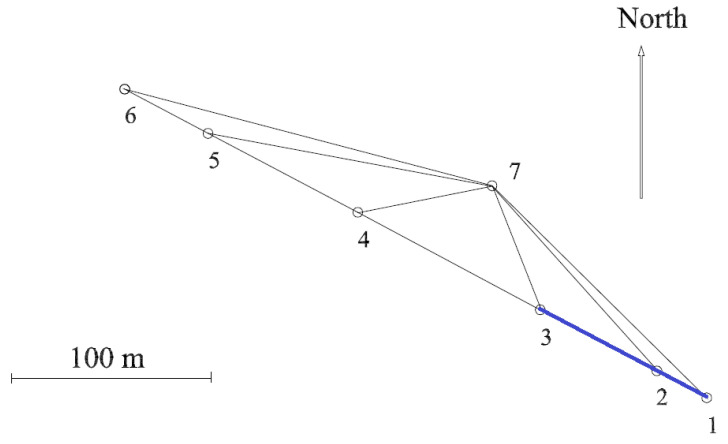
UPV submillimetric calibration test field.

As shown in Figure 10, the UPV metrological infrastructure is a triangle-shaped test field with seven observation pillars which includes an Heerbrugg-type EDM calibration baseline [12]. Oriented north–west, the calibration baseline consists of six observation pillars (No.1 to No.6) approximately in line at 0, 28, 94, 198, 282 and 330 m.

The pillars have a diameter of 22 cm and height 1.20 m above ground level made of two insulated steel pipes. The inner one covers a concrete structure with an square metre foundation that extends to a depth of 60 cm. The outer steel pipe prevents the inner pillar from differential dilations due to meteorological effects. To install measuring instruments on the top they have a double forced-centring mount system: the standard 5/8” fixing screw and a Kern-type trivet system.

Three measurement campaigns were carried out from December 2007 to February 2008 using the following total stations: Topcon GTS605, Leica TPS 1200+ and Leica TDA5005. All of them proved to be compatible within a combined least squares adjustment and the coordinates given in Table 1 were adopted as first set of approximate local coordinates. The coordinates are referred to a local right handed Cartesian system whose origin is the top of the pillar No.1, the *x*-axis points to pillar No.6 and *z*-axis is positive upwards.

The horizontal and vertical stability of the pillars over time has been periodically monitored [45,46,47]. Since no significative displacement was detected, this first set of coordinates have been safely regarded for geometrical reduction computations in subsequent campaigns.

The field campaign to transfer scale from the Nummela Standard Baseline (NSB) in 2012 was carried out from 28 May to 1 June by the Finnish Geospatial Research Institute (FGI). The measurements were done using the Kern ME5000 No. 357094 of the Aalto University along with the reflector No.374414 which were calibrated at Nummela before and after the transferring campaign. Dry and wet temperatures were measured using calibrated Thies Clima Assmann-Type psycrhometers with an estimated ±0.3 ${}^{\circ }$C uncertainty and air pressure was measured using two calibrated Thommen 3B4.01.1 aneroid instruments with an estimated ±0.3 hPa uncertainty. The EDM and the reflector were installed in the 5/8” fixing screws of the pillars using Leica GDF321 tribrachs. As the observational scheme consisted in four sets of ‘double-in all-combinations’distances, every distance was consequently measured 16 times. This observational scheme was strictly repeated in the subsequent four field campaigns.

Therefore, the traceability chain for the UPV baseline distances that were to be use as ground truth was: SI definition of the metre, quartz gauge bar, Väisälä interferometer, FGI Nummela Standard Baseline, FGI Mekometer ME5000 and UPV calibration baseline. The resulting nominal distances were obtained with expanded total uncertainties $U_{k=2}$ of 0.1–0.3 mm (Table 2).

### 4.2. Description of the Experiment

To be prove accurate, the GNSS-based distances had to be compared with the SI-traced distances provided by the FGI in 2012. Furthermore, since accuracy comprises both repeatability and reproducibility, the comparison had to be done over time with GNSS measurements carried out under different environmental conditions. Nonetheless, the SI-traced distances provided by the FGI were realized by the pillars in a specific epoch (June 2012), and consequently their stability over time has to be considered as a critical part of the study. Considering the construction features of the pillars, errors due to vibrations and reproducibility of the centre mount can be assumed negligible, but the possible instability of pillars, which chiefly depends on the grounding and the geological profile, would diminish the uncertainty of SI-traced distances of reference. The instability of pillars is normally included in the corresponding uncertainty budget [22], but detecting possible pillar displacements at submillimetric level requires special instruments like the out-of-production ME5000 or laser trackers [14,48]. For this reason, the experiment was planned to be coincident with the deformation monitoring campaigns of the UPV calibration baseline which were annually carried out from year 2013 to year 2016. In each campaign, the EDM and GNSS techniques were interlaced in a way that the distances obtained can be assumed to be done at the same epoch. The EDM distances were measured in daytime, while the GNSS observations were collected in 12-hour sessions during nighttime. In the following paragraphs, the operational scheme of both types of measurements are further described.

With regard to the EDM campaigns, an agreement between the UPV and the *Universidad Complutense de Madrid* (UCM) enabled us to use the Mekometer ME5000 No. 357094 along with reflectors No.374447 and No.374448 to carry out the deformation monitoring of the UPV baseline [46]. The dates and instruments used in each campaign are shown in Table 3. Dry and wet temperatures were always measured at both ends by using calibrated Thies 4410 Assmann-Type psychrometers with an estimated ±0.3 ${}^{\circ }$C uncertainty and air pressure was measured using two calibrated Thommen 2A4.611 aneroid instruments with an estimated ±0.3 hPa uncertainty. The EDM and the reflector were installed in the 5/8” fixing screws of the pillars using Leica GDF321 tribrachs. Each campaign consisted in four series following exactly the same observational scheme as the first campaign carried out by the FGI in 2012 to transfer absolute scale from Nummela. Since each series of EDM measurements takes around one day, the total observation time for each campaign is four days. This observational scheme not only provides the actual EDM distances between pillars with uncertainties of 0.1–0.3 mm, but also the zero-error for the set distance meter and reflector, which is determined in accordance to the full procedure of the ISO17123-4.

The raw distances measured in the four field campaigns were processed following exactly the same steps that the FGI scale-transferring campaign in 2012. The three corrections that were sequentially applied were: refraction correction, calibration correction and geometrical reduction.

The information required for computing the refraction correction consists of dry temperature, wet temperature and air pressure collected at both ends of the beam path for every measured distance along with the calibration values of the thermometers and barometers used in the field campaign. The actual refraction index for every single distance is computed using the precise Ciddor and Hill method [49,50] as recommended by the International Association of Geodesy when the scale is required to be better than 1 ppm. It is worth noting, however, that this formulation has an incorrect sign, as recently pointed out by [51]. Once obtained the actual refraction index, the three meteorological corrections recommended for the ME5000 [11] were computed, though only the first speed correction was retained because the second speed correction and the beam correction were negligible even for the longest distance (330 m).

The calibration correction was then applied to the slope distances resulting from the refraction correction. Applying the calibration parameters (zero-error and scale error) improves the accuracy of the measured distances, though the precision of the resulting distances decreases as the uncertainty of the calibration parameters has to be taken into account.

Finally, the geometrical reduction is applied to obtain the distances in Table 4.

Concerning the GNSS campaigns, they were carried out in 12-h static sessions during the nighttime once the daily EDM measurements were finished. Table 5 shows the dates and other details concerning the GNSS campaigns. Since pillars No.1 and No.3 had better GNSS observing conditions than the rest, they were selected to conduct the experiment. Nonetheless, the trees surrounding the baseline had considerably grown from 2013 to 2016, making the multipath error apparent even for the selected pillars. Therefore, we decided to reduce the GNSS data interval from 15 s to 1 s in the 2016 campaign so that the methods to detect and mitigate the multipath error that were being developed at Space Geodesy and Navigation Laboratory of the University College London (SGNL-UCL), which were described in the previous section, could be better implemented and tested.

With regard to the GNSS antennas, we always used the same two 3D choke-ring Leica AR25.R4, which were individually calibrated by the University of Bonn by using the anechoic chamber method [52,53]. The two antennas were set up carefully oriented towards the geodetic north which was rigorously realized by benchmarks that are permanently located near every pillar. The GNSS distances finally obtained are given in Table 6. It is worth noting that all the DD residuals and testing results in this study are consistent with the solution by Bernese 5.2 [21].

The corresponding differences among the GBDM distances, the ME5000 distances, as well as the original calibrated distance traced to the metre-SI (which is displayed in Table 2) are shown in Table 7.

The GNSS measurements in 2013 were collected using four single-constellation (GPS-only) Trimble 5700 receivers and two signal splitters. They were performed in the daytime shortly before the corresponding monitoring campaign using the UCM Mekometer ME5000. The differences between the computed GNSS distances and their corresponding FGI-certified and UCM-Mekometer ME5000 were respectively +0.12 mm and −0.13 mm. Taking into account their respective uncertainty (standard deviation for GNSS), the difference can be considered statistically negligible. In addition, there is no significant evidence of pillar displacements from 2012 to 2013.

In 2014, the GNSS measurements were collected using two single-constellation Leica GS10 receivers and they were performed in the nighttime whereas the corresponding monitoring campaign using the UCM Mekometer ME5000 was carried out during the same days in the daylight. The comparison between the GNSS distance and the EDM-derived for this epoch yielded a difference of 0.28 mm, which can be considered statistically negligible taking into account their respective uncertainties (standard deviation for GNSS). In addition, non-significant is the baseline length variation in this epoch with respect to the previous epoch. This agrees with the conclusion obtained by a dedicated deformation approach for the entire network [47], which concluded no significant displacements for the baseline.

The GNSS measurements in 2015 were collected in the nighttime using two multi-constellation Leica GS10 receivers. The spantime was eight hours and the receivers measured carrier phase observables for the following number of satellites: 21 GPS, 19 GLONASS and 4 Galileo. The corresponding monitoring campaign using the UCM Mekometer ME5000 was carried out during the same days in the daylight. Since both EDM-based distances, i.e., FGI-certified and the one obtained by using the UCM Mekometer ME5000, agreed well, it is also concluded that there were no significant displacements. Once again, the difference between the obtained GNSS distance and the corresponding using the UCM Mekometer ME5000 is satisfactorily small.

Similarly, the GNSS measurements in 2016 were collected in nighttime by using two multi-constellation Leica GS10 receivers whereas the measurements using the UCM Mekometer ME5000 were carried out during the same days in daytime. No significant discrepancies could also be found among any of the types of distances.

Nevertheless, all these distances constitute but a limited sample so that additional measurements need to be made to draw definitive conclusions and further research has to be conducted in order to assess the validity of this degree of agreement and possibly reduce the standard deviation of the GNSS distances computed.

## 5. Conclusions and Future Work

We have proposed and tested new methods to develop a submillimetric GNSS-Based Distance Meter (GBDM) for application to length metrology in open air for distances up to 1 km, as an alternative to more expensive approaches to the problem like two-colour telemeters or laser trackers, which are currently under development. We present the corresponding functional model as well as specific methods to mitigate the main source of error in the GBDM determination of length for baselines up to 1 km: the multipath error. Consistency with the unit of length (SI-metre) has been shown within few tenths of a millimetre by using a calibration baseline of reference having distances traced to the SI-metre and a ME5000 distance meter.

The future application of the GBDM to longer baselines, in the range of 1 to 5 km, can be expected as soon as some difficulties are circumvented: mainly, the correction and corresponding assessment of uncertainty of the impact on the baseline length of the residual tropospheric delay, which should not be neglected especially in the case of relatively large height differences. The evaluation of different absolute antenna calibration models for use in the problem at hand and the corresponding uncertainty assessment is also a question to be addressed in the future.

The present study has been realized with the main focus on the GPS constellation, and the marginal use of satellites from other constellations (Galileo and GLONASS). An optimized, fully multi-constellation, approach needs also to be further developed.

## Figures and Tables

**Table 1:** Set of approximate 3D local coordinates (2008) and their standard deviations. The third coordinate *z* refers to the height at the top of the pillar relative to the top of pillar 1.

Pillar	*x*(m)	$\sigma_{x}$ (mm)	y(m)	$\sigma_{y}$ (mm)	z(m)	$\sigma_{z}$ (mm)
1	0.0000	0.0	0.0000	0.0	0.0000	0.0
2	28.3780	0.4	0.1603	2.7	0.0187	0.3
3	94.3948	0.4	0.1118	1.9	0.2010	0.3
4	198.0027	0.4	0.1322	2.1	0.2060	0.4
5	282.7853	0.4	0.0683	3.2	0.4489	0.4
6	330.0030	0.4	0.0000	0.0	0.3129	0.4
7	144.8110	0.5	43.4056	1.8	0.0424	0.3

**Table 2:** Calibrated slant distances traced to SI-unit metre through the Nummela Standard Baseline and their expanded uncertainties *U* (*k* = 2).

Line	Distance (m)	$U_{k=2}$ (mm)
1–2	28.38334	0.20
1–3	94.40121	0.21
1–4	198.00786	0.26
1–5	282.78960	0.30
1–6	330.01110	0.33
1–7	151.17235	0.24
2–3	66.01886	0.21
2–4	169.62493	0.24
2–5	254.40681	0.29
2–6	301.62825	0.31
3–4	103.60708	0.22
3–5	188.38847	0.25
3–6	235.61008	0.28
3–7	66.59225	0.20
4–5	84.78211	0.21
4–6	132.00335	0.23
4–7	68.57778	0.19
5–6	47.22197	0.20
6–7	190.22249	0.24

**Table 3:** Dates of the four campaigns and results of the calibration process for the Kern RMO5035 reflectors used. All values in mm.

Year	Dates	ME5000 SN	Frequency (MHz)	Reflector SN	Zero-Error (mm)	σ (mm)
2013	25 June–28 June	No.357094	479.35783	No.374448	0.1509	0.0166
2014	24 June–27 June	No.357094	479.35778	No.374447	0.1079	0.0146
2015	21 July–24 July	No.357094	479.35776	No.374447	0.0868	0.0183
2016	12 November–16 November	No.357094	479.35773	No.374448	0.1224	0.0133

**Table 4:** Electronic Distance Meter (EDM) distances obtained in years 2013–2016 and their corresponding standard deviation and expanded uncertainty *U* (*k* = 2).

Year	ME5000 Distance	σ	$U_{k=2}$
2013	94.40146 m	0.08 mm	0.21 mm
2014	94.40057 m	0.09 mm	0.22 mm
2015	94.40108 m	0.08 mm	0.21 mm
2016	94.40093 m	0.07 mm	0.21 mm

**Table 5:** GNSS instruments used in every campaign and time interval of data registration.

Year	Dates	Sessions	GNSS Receivers	GNSS Antennas	Interval (s)
2013	25 Jun–28 Jun (day)	4	Trimble 5700	Leica AR25 R4	15
2014	24 Jun–27 Jun (day)	4	Leica GS10	Leica AR25 R4	15
2015	21 Jul–24 Jun (day)	4	Leica GS10	Leica AR25 R4	15
2016	12 November–16 November (day)	4	Leica GS10	Leica AR25 R4	1

**Table 6:** GNSS-Based Distance Meter (GBDM) distances obtained in years 2013, 2014, 2015 and 2016, and their corresponding standard deviations.

Year	GNSS Distance	σ (mm)
2013	94.40133 m	0.036 mm
2014	94.40085 m	0.034 mm
2015	94.40110 m	0.040 mm
2016	94.40126 m	0.052 mm

**Table 7:** Differences between the ME5000, SI-traced and GNSS distances, and their corresponding expanded uncertainties *U* (*k* = 2).

Year	ME5000-SI	$U_{k=2}$	GNSS-SI	$U_{k=2}$	GNSS-ME5000	$U_{k=2}$
2013	0.25 mm	0.30 mm	0.12 mm	0.42 mm	−0.13 mm	0.42 mm
2014	−0.64 mm	0.30 mm	−0.36 mm	0.40 mm	0.28 mm	0.40 mm
2015	−0.13 mm	0.30 mm	−0.11 mm	0.45 mm	0.02 mm	0.45 mm
2016	−0.28 mm	0.30 mm	0.05 mm	0.54 mm	0.33 mm	0.54 mm

## Data Availability

All data included in this study are available upon request by contact with the corresponding author.
